# Systemic risk from investment similarities

**DOI:** 10.1371/journal.pone.0217141

**Published:** 2019-05-23

**Authors:** Danilo Delpini, Stefano Battiston, Guido Caldarelli, Massimo Riccaboni

**Affiliations:** 1 Dept. of Economics and Business, Università degli Studi di Sassari, Sassari, Italy; 2 IMT Institute for Advanced Studies, Lucca, Italy; 3 Dept. of Banking and Finance, University of Zurich, Zurich, Switzerland; 4 ISC-CNR Uos “Sapienza”, Rome, Italy; 5 Dept. of Managerial Economics, Strategy and Innovation, KU Leuven, Leuven, Belgium; Universidad Veracruzana, MEXICO

## Abstract

Network theory proved recently to be useful in the quantification of many properties of financial systems. The analysis of the structure of investment portfolios is a major application since their eventual correlation and overlap impact the actual risk by individual investors. We investigate the bipartite network of US mutual fund portfolios and their assets. We follow its evolution during the Global Financial Crisis and study the diversification, as understood in modern portfolio theory, and the similarity of the investments of different funds. We show that, on average, portfolios have become more diversified and less similar during the crisis. However, we also find that large overlap is far more likely than expected from benchmark models of random allocation of investments. This indicates the existence of strong correlations between fund investment strategies. We exploit a deliberately simplified model of shock propagation to identify a systemic risk component stemming from the similarity of portfolios. The network is still partially vulnerable after the crisis because of this effect, despite the increase in the diversification of multi asset portfolios. Diversification and similarity should be taken into account jointly to properly assess systemic risk.

## Introduction

The Global Financial Crisis (GFC) of 2007-2008 has highlighted the systemic risk stemming from the increasing interdependence both between large institutional investors and among global assets [1–3]. On the one hand, large institutional investors allow for a better diversification of individual risk: the larger the number of different assets in a portfolio, the smaller the fraction of an idiosyncratic shock an investor has to bear. On the other hand, the GFC has shown that cross-sectional dependencies between assets can cause idiosyncratic shocks (i.e. related to the distress/bankruptcy of a single specified asset) to spread, ultimately threatening the stability of the entire financial system. According to modern portfolio theory, risk depends on the share of individual stock holdings and the variance–covariance matrix among its holdings [4]. Hence, theoretical models imply that a portfolio should be properly diversified to reduce risks (unsystematic risks), but how to construct a well-diversified portfolio when multiple investors are simultaneously active on the market still remains not fully understood. There is consensus in the literature that a strong risk reduction of holdings can be realized by increasing the number of assets in a portfolio [5–8]. Indeed, between 1997 and 2012, assets in the equity, balanced, and fixed income mutual funds have increased by more than 400 percent [9]. The efficacy of diversification strategies depends, however, on market conditions and systemic risk that may moderate the relationship between diversification, fund performance, and risk. Recently, it has been shown that the benefit of diversification increases in high market volatility conditions, such as the GFC of 2007-2008, meaning that the number of stocks needed to achieve a well-diversified portfolio increases under those market conditions [10]. Unfortunately, the implications of such an increase of portfolio diversification during crises is still unclear. Moreover, the empirical evidence accumulated during the GFC has raised legitimate doubts on the effectiveness of portfolio diversification strategies to reduce risk [11].

Recently, systemic risk in financial systems has been increasingly investigated through the lens of network theory [1–3, 12–21]. Most of the work so far has focused on the network of interbank loans, even though there is a paucity of data about the real-world structure of financial networks. Interestingly, it has been found that, when the magnitude of negative shocks is large and the network is scale-free, a more densely connected financial network (corresponding to a more diversified pattern of investment) serves as a mechanism for the propagation of shocks, leading to a more fragile financial system, thus increasing systemic risk [14].

Even though a better diversification may reduce risk for an individual portfolio, the structure of the similarities across mutual funds could be a key element of systemic risk [22]. Depending on that structure, on the way diversification is pursued and the level of interdependence between investment strategies, increasing portfolio diversification during a crisis might possibly increase the cross-correlation among assets thus amplifying systemic risk. Therefore, the exact role played by the evolution of the financial network during crisis in potentially creating systemic risk remains, at best, imperfectly understood. The same holds for the role of global fund managers which appears to have been studied mostly in simulated scenarios [23]. We contribute to the literature by analyzing, through the lens of network theory, the bipartite network of US mutual funds over time and throughout the GFC as well as in simulated scenarios.

The fund holding network has been considered previously in [24] where the correlation between changes in a firm’s position in the network and future stock market performance is considered, and in [25] where the CRSP database was studied to detect possible conflicts of interest in the strategies of multi-fund managers. In [26] the size distribution of funds has been investigated in detail, while in [27] a size-dependent model of fund growth has been proposed to explain its shape. The existence of a positive flow-performance relationship and fire-sales have been documented for mutual funds [11]: investors tend to redeem their investments in response to negative shocks. In some circumstances, overlapping portfolios and asset liquidations can force mutual funds to sell additional assets potentially triggering cascading effects in the market [22, 28].

In this paper we consider bipartite networks of portfolio holdings [1, 15, 16] with the intent to provide a better understanding of the relationship between the similarity of individual investment strategies and systemic riskiness. We use the network of US equity mutual funds as a test case. We exploit a deliberately simple model of distress propagation and stress test the network to study how the systemic fragility of the system depends on the overlap between portfolios due to correlated investment strategies. This is done through simulations and comparison with null models of random versus correlated investments. Finally we make an assessment of how much network vulnerability has changed across the Global Financial Crisis.

## Materials and methods

### Dataset

The US Mutual Fund market is the largest in the world: with 15$ trillion assets under management at year-end 2013, it accounts for about half the total value in mutual fund assets worldwide. In this study, we analyse data from the Survivor-Bias-Free Mutual Fund Database provided by Chicago Booth Center for Research in Security Prices (CRSP). It includes open-ended mutual funds registered with the Securities and Exchange Commission and provides detailed information about the composition of managed portfolios. A mapping of funds to the portfolios of assets they manage is provided and detailed information about portfolio holdings, including the market value of each holding, is delivered on a quarterly basis. In the following, we provide a formal construction of the bipartite network of holdings. We also present a schematic description of the model of distress propagation considered in the main text. Access to the data requires a valid (non-free) subscription that can be acquired issuing a subscription enquiry to the CRSP center.

### Bipartite graph of portfolio holdings

**Fig 1:**
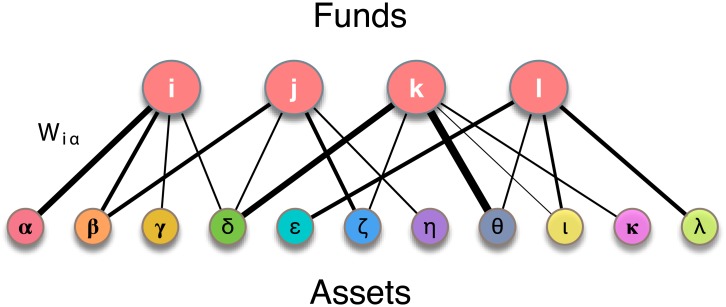
Graph of portfolio holdings. The network of portfolio holdings can be represented as a bipartite graph. The two vertex classes are the *funds* {*i*, *j*, *k*, …} and the *assets* {*α*, *β*, *γ*, …} in their portfolios. Each edge (*i*, *α*) represents a specific holding relationship. The edge weight *W*_*iα*_ is equal to the total market value of security *α* owned by fund *i* in its portfolio.

We represent portfolio holdings in terms of a bipartite graph [16]. The two vertex classes are the set of mutual funds *i* = 1, …, *N*_*f*_, and the set of the different assets *α* = 1, …, *N*_*a*_ in their portfolios. The degree *k*_*i*_ of vertex *i* is exactly the number of distinct assets held in the portfolio of fund *i*. Edges incident at vertex *i* can be assigned weights *W*_*iα*_ equal to the total market value of the shares of asset *α* and the graph is also conveniently represented by a *N*_*f*_ × *N*_*a*_ matrix *B* with elements *B*_*iα*_ = *W*_*iα*_. We indicate as *G*(*N*_*f*_, *N*_*a*_), or simply *G*, the undirected bipartite network of portfolio holdings. If we wanted to retain just the topological information of which asset is owned by which fund, we could define an unweighted graph *G*^0^(*N*_*f*_, *N*_*a*_). This would correspond [29, 30] to the incidence matrix *B*^0^ whose elements $B_{i\alpha }^{0}$ are 1 if asset *α* is in the portfolio of fund *i* and 0 otherwise. A schematic picture of the network of holdings is provided in Fig 1.

The *size* of fund *i* is given by

$$\begin{array}{c} S_{i}=\sum_{\alpha =1}^{N_{a}}W_{i\alpha }\;, \end{array}$$

where it is understood that *W*_*iα*_ = 0 if asset *α* is not in the portfolio. We clearly see that, in network terms, fund size corresponds to *node strength* [31, 32]. The sum *S*_*tot*_ = ∑_*i*_ *S*_*i*_ represents the *total value* of the system.

The quantities *S*_*i*_ and *W*_*iα*_ are expressed in currency units. We also define the *portfolio weights* *w*_*iα*_ = *W*_*iα*_/*S*_*i*_, that represent the fraction of portfolio wealth corresponding to each asset. The indices of diversification and similarity discussed in the main text are all expressed in terms of the *w*_*iα*_s.

Due to portfolio reallocations, the set of assets in a portfolio and the edge weights can change in time; also the number of indexed funds in the dataset depends on time. Reporting of portfolio reallocations by funds are not synchronous and some choices are due in order to aggregate the information about holdings over a given time frame and construct the graph representing the system. Since portfolio composition is reported on a quarterly basis, in our analysis we choose a three-month time frame for aggregation. We create quarterly snapshots of the bipartite network by means of the following procedure. For each quarter, we consider the set of the funds *i* = 1, …, *N*_*f*_ for which a mapping to a portfolio exists. For the given quarter and for each fund *i*, we retrieve the holding information at the most recent report date *t*_*i*_. Basically, this information is given by the set of assets in the portfolio and the corresponding market values. Fund *i* is inserted into the graph for the current quarter and so are the links (*i*, *α*), with *α* ∈ [1, *N*_*a*_], for all assets held by the fund. Each link is assigned weight *W*_*iα*_ equal to the market value of the holding it represents.

When parsing the holding relationships in the dataset, attention has to be paid to the issue of *fund classes*. As a matter of fact, a fund may issue different types of shares all corresponding to the same underlying portfolio. In the database, different classes of a funds are associated to different unique identifiers, as if they were distinct funds. For the purpose of our analysis, we consolidated information about fund classes, to avoid including the same fund multiple times.

### A basic model of distress propagation

Let us suppose that during period *t* the prices of some stocks undergo a negative shock *δ*_*α*_(*t*) = [*v*_*α*_(*t*) − *v*_*α*_(*t* − 1)]/*v*_*α*_(*t* − 1) < 0, where *v*_*α*_(*t*) is the market price of *α*. These downward moves produce a negative variation in the values of the portfolios that hold those stocks. As a consequence, we expect individual fund investors to redeem their shares, eventually forcing the asset manger to sell assets in order to meet the requests of redemption. Generally, we expect a larger (relative) drop Δ_*i*_ of the total net assets of fund *i* to correspond to a higher probability for an investor to redeem her shares of the fund. Let *V*_*α*_ be the total market value of *α* disinvested in the process of asset selling. The instantaneous increase in the offer of *α* will determine a negative impact in its price on the market and a new drop *δ*_*α*_(*t* + 1) = λ[*V*_*α*_(*t* + 1)], where the function λ is called the *price impact* (deterministic or stochastic). We expect the structure of similarities between real portfolios to be reflected also in the actions taken by agents (i.e. asset managers) in response to a shock. Accordingly, we enrich the above dynamics including a heterogeneous probability for agents to mimic the activity of funds that have already liquidated part of their portfolio. More precisely, a fund *i* that reacts to a negative shock may imitate the behavior of the most similar fund *j* that has already reacted. This occurs with probability given by the value of their cosine similarity. Imitation consists in the fact that *i* would liquidate only the stocks liquidated by *j* that are in both portfolios.

In the absence of large random effects and portfolio reallocations, such a recursive dynamics will bring a progressive reduction of the total value of the system. In the literature, the price impact of asset liquidations has been modeled as a function of average daily volume traded and asset’s price volatility [16]. For the sake of simplicity, in our analysis we assume the impact on the price of security *α* to be a linear function of the traded value *V*_*α*_. More precisely we take

$$\begin{array}{c} \lambda \left(V_{\alpha }\right)=\frac{V_{\alpha }}{V_{\alpha ,tot}}\;, \end{array}$$

where *V*_*α*,*tot*_ is the total value of stock *α* owned by funds at the beginning of each trading period. The values *V*_*α*,*tot*_ can be considered as proxies of stock liquidity [33, 34]. In this sense, the previous choice for λ accomodate for heterogeneity in stock liquidity characteristics. The simulations discussed in the next section also assume that fund managers liquidate a fraction of portfolio holdings equal to the relative drop of total net assets. When a fund’s trading occurs through imitation of another fund’s liquidations, we also assume that the imitating fund can liquidate only common assets. In this case, it is not allowed to trade more than the value accounted for by such assets. Results refer to a negative shock of −30% to ten most common assets in fund portfolios. This shock is propagated for a number of periods and the percentage total loss of value of the system is computed for each one.

Such a model provides an admittedly simplified representation of shock propagation within a bipartite network of portfolios and their holdings, which incorporates *contagion effects* due to common exposures of overlapping portfolios. It should be considered a very crude approximation of the dynamics in the time span that divides subsequent reallocations executed by fund managers, wherein the network can be regarded as static.

It is worth noting that we model a system where agents correspond to asset managers. Redemption requests by individual fund investors act as a driver in transmitting shocks but their behavior is not modeled explicitly. In future work a fully fledged agent based model of asset management can be develop to accommodate more sophisticated behavioral assumptions and market features.

## Results and discussion

As discussed in the previous section, a network of portfolio holdings is conveniently represented as a bipartite graph where the two vertex classes are the set of portfolios and the set of the assets they hold, respectively. For our purposes, we make the identification of funds with the portfolios they manage.

Within this framework, we consider the usual notion of diversification of an individual portfolio. This depends on the number of different assets in the portfolio, as well as on their weights and on the correlation between asset price variations. Diversification reduces the average loss that a portfolio undergoes when a random idiosyncratic shock hits one of its holdings. It is a widely accepted and employed strategy to mitigate the effects of market risk at the portfolio level. Then we add a second dimension to the discussion, by studying the *similarity* of two portfolios, also known as asset overlap [11]. The opposite of similarity is a measure of the difference between two portfolios and we may name it “differentiation”, to distinguish such notion from the notion of portfolio diversification.

The analysis is performed with an attention to the evolution of the network throughout the GFC. One event that has marked the escalation of the crisis was the UK investors queuing to take their money out of Northern Rock in September 2007 and, as it is well known, the peak of the crisis has been reached in September 2008, culminating in the collapse of Lehman Brothers. To make comparisons of the network at different times, we focus on three reference quarters, 2006Q3, 2007Q3, 2008Q3.

### Diversification and similarity

It is widely understood that diversification can reduce unsystematic risk, this wisdom dating back to the studies of Markowitz on portfolio diversification. However, less well understood is the optimal diversification strategy when multiple investors are simultaneously active on the market with possibly similar, or otherwise correlated, investment strategies [35]. In this paper we consider diversification and overlap between portfolios as related notions. We make the hypothesis that, while diversification reduces risk for an individual portfolio, the similarity of portfolios can be a source of systemic risk. There can be multiple ways to attain the same level of diversification for a given portfolio. And this is true independently of how the other portfolios invest their money. It can not be excluded that, depending on the topological properties of the holding network, on the criteria of selection of new holdings and how the decisions by different portfolio managers depend on each other, a raise in diversification may increase portfolio overlap and translate into a raise of systemic riskiness.

**Fig 2:**
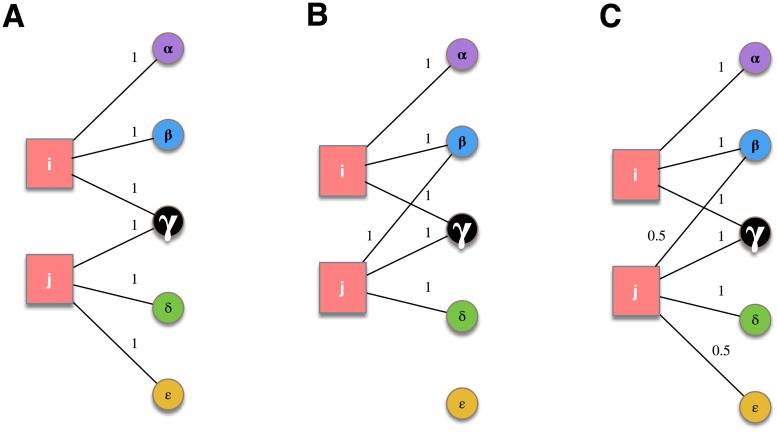
Three case studies, with the same values of the portfolios (*S*_*i*_ = *S*_*j*_ = 3) but different values of the inverse Herfindahl-Hirschmann index and of the cosine similarity between them.

We support these conjectures by first considering three illustrative cases for a stylized network of holdings. They are built on top of the graphs depicted in Fig 2, with two funds *i*, *j* investing in five different assets *α*, *β*, *γ*, *δ*, *ϵ*. We are interested in the systemic effect of a negative external shock impacting the market value of an asset owned by funds. To this aim we use a deliberately simplified toy model of dynamics to keep the discussion as schematic as possible and consider the effects of similarity and portfolios diversification separately. We will make the following assumptions:

1. All assets have unit value at first;
2. A portfolio manager liquidates the whole position as a response to the negative shock, such as in a scenario of extreme flow-performance relationship;
3. When assets are disinvested by a fund, this determines a proportional decrease in the market value of the asset.

We measure diversification by the *inverse of the Herfindahl–Hirschmann index*. This is defined as

$$\begin{array}{c} h_{i}={\left[\sum w_{i\alpha }^{2}\right]}^{-1}. \end{array}$$

where *w*_*iα*_ is the weight of asset *α* in the portfolio of fund *i*. The Herfindahl–Hirschman index is a typical measure of concentration [36–38] and its inverse can be regarded as the number of leading assets in the portfolio. The value of *h*_*i*_ will be close to one for a fund that invests primarily in a single asset; the opposite case is represented by a uniform investment, with the same fraction of portfolio wealth invested in each asset, when we have that *h*_*i*_ is equal to the degree of fund *i* in the network.

To measure the overlap between two portfolios, we adopt *cosine similarity* [11]

$$\begin{array}{c} s_{ij}=\frac{\sum w_{i\alpha }w_{j\alpha }}{∥{\vec{w}}_{i}∥\;∥{\vec{w}}_{j}∥}. \end{array}$$

where $∥{\vec{w}}_{i}∥$ stands for the Euclidean norm of the vector ${\vec{w}}_{i}$ of the portfolio’s weights. Portfolio similarity depends indeed on two factors: the number of assets they have in common and the similarity of the weights attached to common assets. This index is equal to one for two portfolios that contain exactly the same assets in exactly the same proportions. It will be smaller than one otherwise, and equal to zero for two portfolios that do not overlap at all.

For the illustrative examples of Fig 2, we assume that the value of asset *γ* goes to zero due to an external shock and either one of the two funds reacts by liquidating the whole portfolio and quitting the market. The portfolios have equal value (or “size”), *S*_*i*_ = *S*_*j*_ = 3, and the total value of the system is the sum of the sizes. Here, we define systemic damage as the percentage variation in total value after portfolio liquidation and subsequent reduction in asset values.

#### Case A

The two portfolios are equally diversified, with *h*_*i*_ = *h*_*j*_ = 3, and their similarity is *s*_*ij*_ = 1/3. Fund *i* reacts to the shock liquidating its portfolio and quits the market. Asset *γ* is the only one shared with fund *j* and the systemic damage amounts to Δ_*A*_ = −2/3 ≃ −67%.

#### Case B

In this scenario *j* prefers to invest one unit of value in *β* instead of *ϵ*. In doing so, it attains the same diversification as in case A but mimics *i*’s investment strategy more closely. This results in double the similarity of case A, *s*_*ij*_ = 2/3. When *i* liquidates its portfolio, it sells one unit of *β* and, as a consequence, the value of *β* decreases by one half. This provokes a corresponding reduction in the value of *j* and the systemic damage is now $\Delta_{B}=\frac{(1/2+1)-6}{6}=-\frac{3}{4}≃-75\%<\Delta_{A}$.

#### Case C

With respect to case A, *j* attains higher diversification with a holding of 0.5 of *ϵ* and investing the other 0.5 on *β*: $h_{j}=\frac{18}{5}≃3.6$. In this particular case, the increase in diversification causes an increase of similarity as well, $s_{ij}=\frac{1/2+1}{\sqrt{3}\;\sqrt{5/2}}≃0.55$. After portfolio liquidation by *i* the value of *β* reduces to 1/3 and we get $\Delta_{C}=\frac{1}{6}(\frac{1}{2}\times \frac{1}{3}+1+\frac{1}{2})-1=-\frac{13}{18}≃-72\%<\Delta_{A}$.

To summarize, the above toy examples highlight the following aspects:

1. Case B exemplifies that for a fixed level of diversification a higher level of similarity between portfolios can lead to a riskier system in the presence of feedback effects;
2. Case C exemplifies that higher diversification may be obtained to the cost of an increase in portfolio similarity, again making the system riskier.

In cases B and C the increase of overlap is direct consequence of the fact that, in network terms, the *degree* of *β* has increased by one. That makes the system more fragile also because an initial shock to *β* would have a wider outreach that it had in case A. However, in our example a shock is given only to *γ*. So the degree of the common asset *β* plays a role only in the second stage, where it acts as the medium that transmits to fund *j* the effects of the liquidation of the other fund’s portfolio.

### The network of U.S. equity funds

In this section we investigate diversification and similarity of real portfolios, considering the case of US mutual fund portfolios. Holding information was aggregated as detailed in section Materials and methods. Summary statistics for this network over the considered years are reported in Table 1.

**Table 1:** Summary statistics of the bipartite network of holdings.

Quarter	*N*_*f*_	*N*_*a*_	$\tilde{h}$	*S*_*tot*_ ($B)
2005Q3	1875	6963	42.4	1950.9
2005Q4	1749	6739	44.1	2248.0
2006Q1	1976	7272	44.3	2457.5
2006Q2	2437	9537	43.2	2654.2
2006Q3	2458	9602	42.9	2684.7
2006Q4	2552	10796	43.2	2900.9
2007Q1	2448	11405	42.8	2712.8
2007Q2	2813	11048	43.3	3331.8
2007Q3	2698	14340	43.1	3332.7
2007Q4	2608	20142	45.6	3638.1
2008Q1	2902	26319	51.1	4174.4
2008Q2	3555	27135	49.7	4332.6
2008Q3	3854	26366	45.9	3299.1
2008Q4	3991	29917	46.7	2621.1
2009Q1	3984	30554	51.0	3314.6
2009Q2	4056	31702	53.3	3832.7
2009Q3	3992	30963	53.5	4377.0
2009Q4	4061	29989	52.8	4515.8
2010Q1	3990	30761	50.1	4594.3
2010Q2	3814	30437	53.7	4365.8

Number of funds *N*_*f*_ and assets *N*_*a*_, median of the inverse Herfindahl-Hirschmann index $\tilde{h}$ and total value of the funds in billion dollars.

**Fig 3:**
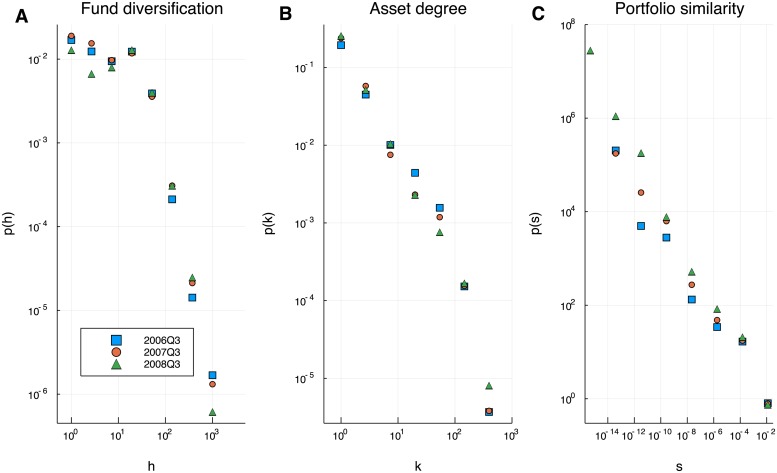
Probability distributions. Probability density functions of the inverse Herfindahl–Hirschmann index *h* (**A**), asset degree *k* (**B**) and cosine similarity *s* (**C**) for three reference quarters. The network is heterogeneous and all the distributions are broad and fat tailed. Most portfolios have few dominant assets but there exist some that have thousands; most assets belong to few funds, but some are extremely popular. Similarities extend over more than 10 orders of magnitude and there exist pairs of almost identical portfolios. Only values *s* > 0 have been considered to compute the histogram.

The network of US funds holdings is heterogeneous in several respects. For instance, the distribution of portfolio sizes is known to be highly skewed and fat tailed. Evidences are presented in [27] in favor of a log-Normal distribution. Of major interest to our analysis are the distributions of diversification, asset degree and portfolio similarity, which we show in Fig 3 for the three reference quarters. In network terms, the *degree* of an asset represents the number of funds that holds it. We see that the popularity of assets in fund portfolios varies over a broad range: many assets are in the portfolios of few funds, but there are also assets that are owned by hundreds or thousands of funds. The distribution of *h* (first panel of Fig 3) decays slowly and extends over several orders of magnitude. Most funds manage portfolios with just few leading assets, the median value $\tilde{h}$ stays between 40 and 50 (see Table 1), but still we see that some funds invest in thousands of different stocks. Between the two extremes, a wide spectrum of intermediate investment strategies exists. The larger the value of *h*, the more the portfolio can be considered diversified. As discussed previously, the definition of *h* is independent on the likeness of different portfolios, while the latter aspect is of major interest in a systemic perspective. The probability density functions of the cosine similarity across portfolios are shown in the third panel of Fig 3. The network is extremely heterogeneous in this respect, with a probability density that spans more than 10 orders of magnitude. Interestingly, the existence of a non negligible probability for similarities close to one points to correlations between investment strategies.

### Comparison with benchmark models of random investments

The real network is heterogeneous with respect to portfolio diversification and asset popularity and exhibits extreme values of both quantities. It is therefore important to assess if large values of portfolio similarities may originate simply from finite size effects (limited number of possible different holdings) or from the presence of very popular assets. The latter would correspond to vertexes with anomalously large connectivity (“hubs” in network terms). We analyze the level of heterogeneity and information content in the network of holdings by comparing the structure of the real network with benchmark models obtained through randomization of the original investments by means of two alternative strategies. Both schemes preserve the number and values of the original holdings of each portfolio but holdings are reassigned to potentially different assets in a random fashion. See also [22] for a similar approach.

In the first case, the new assets are taken randomly with uniform probabilities. We refer to this case as a “random holdings” (RH) model. For holdings of unitary value, it would be equivalent to a bipartite version of an Erdös–Renyi random graph with a constrained degree sequence for funds. In the second case, assets are reassigned through degree-preserving randomization (DPR). We notice that the funds’ degree sequence is preserved by both schemes, while the assets’ is preserved only by the DPR model.

**Fig 4:**
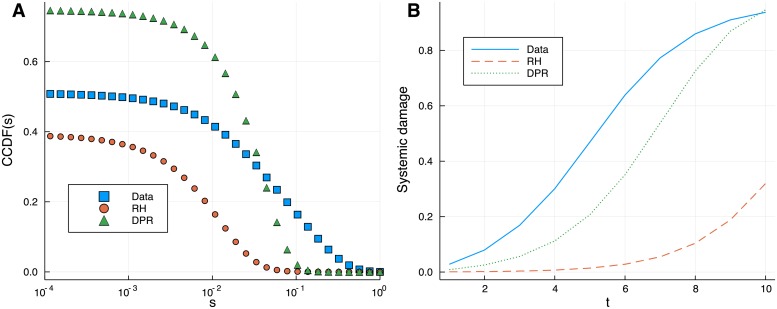
Similarity and systemic risk: Comparison to the null models. Complementary cumulative distribution functions of the similarity for a snapshot of the real network (2006Q3) and the random benchmarks RH and DPR (**A**); the same comparison is performed for the system’s riskiness measured by the total percentage loss ensuing from the propagation of a negative shock of -30% in the value of the 10 most common stocks in the network (**B**). Large values of the similarity between portfolios are more likely in the real world than in the randomized networks even when the degree sequence of assets is preserved. The real network appears as the most fragile. Such fragility is not explained by the role of very popular assets, as shown by the comparison with the the DPR case.

In the left panel of Fig 4 we show the complementary cumulative distribution function *CCDF*(*s*) of the similarity for a snapshot of the real network corresponding to quarter 2006Q3 (the plot does not show the probability mass at *s* = 0). This is compared with its counterparts from the random benchmarks. The *CCDF*(*s*) gives the probability of finding a value of the cosine similarity that is greater or equal to *s* and allows to evaluate differences in the probabilities of very large similarities. In the RH model, the probability decays most rapidly and we see that values of *s* > 0.05 are virtually absent. In the DPR case, the probability of intermediate values of *s* is much larger than in the RH case. Hubs within the assets are not present in the RH models but they are in the DPR case, because there are some in the real case and asset degree is preserved by the DPR scheme. Still, we find that high similarity is unlikely to occur even in the DPR model. In particular, we can find pairs of almost identical portfolios only in the real case. It can be noticed that the probability of low to intermediate values is much larger in the DPR model than in the real network. This suggests that the real network is highly clustered, with groups of similar portfolios and small similarities across different clusters. In this case, we can expect that the rewiring procedure used in the DPR case strongly alters the clustered structure, rearranging holdings in a way that makes original clusters more similar to each other at the expanses of a smaller overlap within each one.

We conclude that the similarities observed in the real network are much larger than can be expected by chance and, more interestingly, they can not be explained with the existence of “hubs” among the assets. Many different mechanisms have been suggested in the literature to account for such a high degree of similarity across portfolios including connections between mutual fund managers and corporate board members, herding behavior and imitation of successful diversification strategies.

The original values of *h* are preserved by the randomization procedures and we can exploit the benchmark models RH and DPR to also test the hypothesis that portfolio overlap is a major source of systemic risk. To this aim, we consider the basic model of distress propagation discussed in Materials and methods, which describes the transmission through the network of an idiosyncratic shock due to common exposures of portfolios. We assume that a negative shock to the value of some assets spread across the network because individual fund investors redeem their portfolio shares in response to a drop in the value of their fund. This produces a negative feedback on the market value of the assets being sold, which in turn triggers a new round of losses for the portfolios owning those assets. It is, of course, a simplified view of the real network dynamics and it does not consider portfolio reallocations. However, reallocation of mutual fund investments is a slow process which might only partially ameliorate the negative impact of the asset sell-off process. In particular, we expect portfolio reallocations by fund managers to take place on a time scale larger than the one of asset price movements on the market and of buying/selling decisions by an individual investor in the fund.

In this context, we measure systemic fragility by the total percentage “loss of value” of the network. We define the initial shock as a relative drop of -30% in the value of the 10 most common stocks in the network. In the right panel of Fig 4 we show the total loss as a function of time for the real network and the benchmark models. The comparison supports the hypothesis that large portfolio similarities contribute to a large extent to the system’s vulnerability to financial shocks.

The less risky case is a model of random holdings. This is expected as it corresponds to a system that is homogeneous on the asset side. Hubs, which are well known to play as accelerators for shock propagation, are absent here and assets are likely to have similar popularity, close in value to the mean of their degree distribution. The real network is most fragile and such riskiness can not be reproduced by the DPR model either. The damage ensuing from a shock to the most common stocks is large and propagates rapidly. Hubs are present in the DPR case. They correspond to the same assets as in the real network and the targets of the initial shock are the same in the two cases. We conclude again that it is not possible to explain the observed fragility as a pure effect of very strongly connected assets and it must depend on the similarity structure of the real portfolios. This latter fact is a major result of our analysis.

### Systemic risk and similarity across the crisis

**Fig 5:**
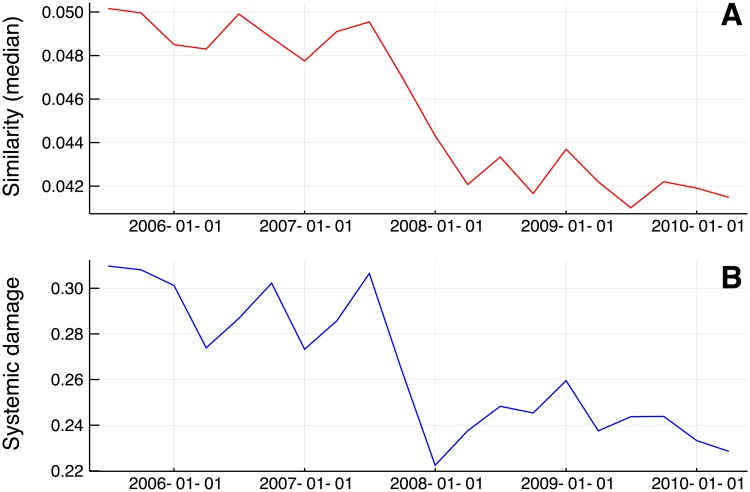
Similarity and systemic damage over time. Median of the similarity *s* (**A**) and systemic damage after *T* = 4 periods (**B**). The similarity across portfolios and the systemic riskiness in the real network are strongly correlated. Both quantities have reduced and the network is less risky after the crisis.

To further support the previous result about the role of portfolio overlap, we compare the level of similarity and vulnerability in the network of US equity funds over the years, see Fig 5. The first is measured by the median of the values of *s* (conditionally to *s* being larger than zero), while riskiness results from simulations of the dynamics introduced earlier over *T* = 4 trading periods.

The similarity of portfolios has decreased during crisis. This agrees with the fast growth in the number of holdings and the holdings/funds ratio over years. This is shown by the summary statistics of Table 1 and it has been recently documented also by using other data sources, such as the Pensions and Investments survey [9]. It is also interesting to note that the median of *h*, also reported in Table 1, has increased as expected during crises, but the number of holdings has increased much more rapidly.

The bottom panel of Fig 5, that shows the evolution of systemic risk with time, exhibits a noticeable resemblance with the evolution of the similarity. We find a value of 0.91 for the correlation coefficient between the two time series and 0.59 for the correlation between their variations. This supports the idea that the level of similarity could be considered as a proxy of vulnerability. The simulated systemic damage has decreased by 22% from 2007Q2 to 2008Q1; we can therefore conclude that the network is more robust after the crisis and this is correlated to modifications of the similarity structure of portfolios.

## Conclusion

We perform an extensive study of the structure and the evolution of the US mutual fund network throughout the Global Financial Crisis of 2007–2008. Even though in normal times households rarely rebalance their retirement saving portfolios [39], it has been found that 21% of them changed investment strategy between February and November 2009 [40]. Such a dramatic recomposition of investment portfolios during crises could have severe consequences on financial stability, even though the role of the asset management industry during the crisis is still questionable. The size of the “ecosystem” of different fund investments has grown steeply over time [9] and, as an average, in the aftermath of the crisis mutual funds have become better diversified and more differentiated. However, simple summary statistics do not tell the whole story. Inspection of the probability density functions shows evidence of a heterogeneous system, with few largely diversified hubs and many specialized funds. Moreover, the probability of the similarity between portfolios decays slowly and large similarities are far more likely that can be expected from benchmark models of a random network of investments. We conclude that a high degree of correlation exists between investment decisions of different funds. This correlation limits the effectiveness of fund of funds diversification strategies.

One of the leading forces behind the emergence of such correlation can be found in the social network of relationships between fund managers [41] and the effects of managerial sharing. Other reasons may be due to herding behavior and the fact that professional investors with similar targets and risk profiles are likely to adopt similar investment strategies. Portfolio managers try to maximize profits and the strategies of many of them will likely include those assets that have proved to be profitable or that can be selected by shared quantitative analysis techniques. Extreme market uncertainty can act as a driver for fund investments during a crisis, when an important fraction of their invested capital is moved from equity mutual funds to fixed-income mutual funds. During the crisis, defined contribution equity mutual funds experienced a large outflow of more that -15%, while flow into the fixed income mutual funds reached a historical peak of +20% [9]. Similarly, many funds might be damaged during crisis at the same time and trigger a second-order effect by which other funds get hit in a failure cascade. In our stylized representation of distress propagation, such second-order effect is induced by fund managers that liquidate portfolios in response to a potentially large number of individual investors that simultaneously redeem their fund shares. However, a complete representation would also consider changes in the network topology as a result of the fact that portfolio managers will try to rebalance their portfolios. Massive co-movements in fund allocations as a response to crisis may have an even higher impact on the market value of securities. This, in turn, may result in significant effects back to the mutual fund network and possibly lead to higher levels of overlap between funds.

Differentiation of portfolios provides a different notion of diversification of investments. Exploiting a stylized model of shock propagation on a simple and static bipartite network, we have shown how strongly portfolio overlap can impact the fragility of the network. We find that the systemic damage in the network is much larger than the damage that can be procured to a network of random investments. By comparison with a degree-preserving random model, we also find that such higher riskiness can not be ascribed solely to the presence of hubs among the assets. In the random benchmarks the diversification of original portfolios is preserved and thus the comparison with the real case provides a way to quantify the systemic risk induced by the similarity of portfolios. We conclude that the Global Financial Crisis has stimulated an increase of diversification but a systemic risk component still exists because of the similarity of investments.

We believe that the evidences presented in our study have implications for both the modeling and the regulation of financial networks. In particular, we show that similarity is correlated with systemic vulnerability. Thus the degree of overlap between the portfolios of large institutional investors should be taken into account for the purpose of assessing systemic risk in holding networks and for devising effective policy actions. In this respect, future research should integrate into our network model more realistic assumptions about the behavior of agents (i.e. asset managers) as well as more detailed information about asset specific market conditions.
